# Increased Serum Level and High Tissue Immunoexpression of Interleukin 17 in Cutaneous Lichen Planus: A Novel Therapeutic Target for Recalcitrant Cases?

**DOI:** 10.1155/2020/6521274

**Published:** 2020-07-24

**Authors:** Magdalena Żychowska, Aleksandra Batycka-Baran, Wojciech Baran

**Affiliations:** ^1^Department of Dermatology, Venereology and Allergology, Wrocław Medical University, 50-238, Poland; ^2^Department of Dermatology, University of Rzeszów, 35-055 Rzeszów, Poland

## Abstract

**Background:**

Interleukin-17 is supposed to play an important role in the pathogenesis of oral lichen planus (OLP). However, there is scarce data in the literature on its significance in the cutaneous variant of the disease.

**Objectives:**

To determine the serum level and tissue immunoexpression of IL-17 in cutaneous lichen planus (CLP).

**Methods:**

Fifty-two adult patients with CLP, without any significant autoimmune or inflammatory conditions, were included in the first part of the study. The control group consisted of 27 age- and sex-matched healthy volunteers. Serum concentration of IL-17 was quantified using enzyme-linked immunosorbent assay (ELISA) kit. In the second part of the study, the tissue expression of IL-17 was assessed in archival paraffin-embedded biopsy specimens from CLP (*n* = 14) against normal control tissues (*n* = 11) using immunohistochemical assays. The expression was evaluated using Zeiss Axio Imager A2 light microscope. Positively stained cells were counted in 10 fields of view for biopsy specimen at 200x magnification, and the mean value was calculated.

**Results:**

The serum level of IL-17 was significantly elevated in patients with CLP, compared with healthy volunteers (0.218 ± 0.221 ng/ml versus 0.126 ± 0.058 ng/ml, respectively; *p* = 0.025). No correlation was found between the serum concentration of IL-17 and patient age, gender, disease duration, extent of skin involvement, the presence or intensity of pruritus, and coexistence of mucosal lesions. In tissue samples from CLP lesions, significantly higher numbers of cells expressing IL-17 were found when compared to a healthy skin (*p* < 0.001).

**Conclusion:**

Elevated serum concentration of IL-17 and high expression in a lesional skin support the hypothesis that IL-17 is implicated in the immunopathogenesis of CLP. These findings may constitute a premise for the future use of anti-IL-17 monoclonal antibodies in the treatment of severe and recalcitrant forms of CLP.

## 1. Introduction

ichen planus (LP) is a chronic immune-mediated dermatosis of a not fully understood aetiology. The disease may involve the skin, mucous membranes, nails, and/or hair follicles and is characterized by a wide variety of clinical manifestations and subtypes [1]. So far, the majority of studies focused on oral lichen planus (OLP). However, due to its distinct clinical course and refractory response to treatment, it is considered by most researchers to be a separate entity. Much less is known about the pathogenesis of cutaneous lichen planus (CLP). The disease is considered by some authors to be the result of delayed hypersensitivity reaction, in which cytokines released by activated T cells attract subsequent inflammatory cells and promote cell-mediated cytotoxicity [2]. Several cytokines, including interleukin- (IL-) 1, IL-6, IL-9, IL-23, tumour necrosis factor-*α* (TNF-*α*), and interferon gamma (IFN-*γ*) were found to be overexpressed in CLP [3]. IL-17 is a pleiotropic proinflammatory cytokine of a key role in the pathogenesis of psoriasis vulgaris and psoriatic arthritis [4]. It is a signature cytokine of T-helper (Th) 17 cells, but it can be also produced by neutrophils, natural killer (NK) cells, mast cells, macrophages, and lymphocytes T *αβ* and *γδ*. IL-17 affects not only the immune cells but also keratinocytes, epithelial cells, and fibroblasts. Its role has also been explored in other skin conditions, including atopic dermatitis [5], alopecia areata [6], bullous pemphigoid [7], systemic lupus erythematosus [8], and neutrophilic disorders [9]. Both serum concentration and lesional tissue expression of IL-17 were found to be increased in OLP [10–21]. On the other hand, there are very few reports in the literature concerning the significance of IL-17 in the cutaneous variant of LP [2, 22–24].

The objective of the study was to evaluate the significance of IL-17 in the pathogenesis of the cutaneous variant of LP. The research was divided into two parts. The first part is aimed at assessing the serum concentration of IL-17 in patients with CLP and correlating it with clinical features, including patient age, gender, body mass index (BMI), disease duration, extent of skin involvement, coexistence of mucosal lesions, and the presence and intensity of pruritus. The aim of the second part of the study was to evaluate the tissue immunoexpression of IL-17 in archival formalin-fixed paraffin-embedded (FFPE) biopsy specimens from 14 cases of CLP and compare the results with those of normal control tissues (*n* = 11).

## 2. Material and Methods

### 2.1. Study Group

In Part I, fifty-two adult patients with histologically confirmed CLP were recruited. All subjects had active lesions on the skin at the time of inclusion. The exclusion criteria were as follows: age under 18, systemic immunosuppressive treatment or phototherapy within two months prior to inclusion, and lichenoid eruption that could be attributed to medication intake and the presence of other relevant inflammatory or immune-mediated conditions (e.g., hypertension, diabetes, allergic diseases, psoriasis, alopecia areata, or rheumatoid arthritis) that could affect the assessment of serum IL-17 concentration. The control group consisted of 27 healthy volunteers from the Regional Blood Donation and Blood Treatment Center, appropriately selected in terms of gender and age. Demographic characteristics of the study group are summarized in Table 1. Detailed medical history with particular interest in patient age, smoking habits, course of LP, and the presence of pruritus was collected with each patient. Thorough physical examination, including mucous membranes, nail apparatus, and scalp inspection, was performed. BMI was calculated as the ratio of weight to height squared and expressed in kg/m^2^. The extent of skin involvement was quantified using the “palm method.” It assumes that the patient's palm surface (not counting the surface of the fingers) corresponds to 1% of the total area of the skin. The clinical subtype of CLP (e.g., papular, hypertrophic, pigmented, linear, and bullous) was determined in each patient, as well. The intensity of itching was assessed using numeric rating scale (NRS), which is an easy and commonly used tool in studies on the severity of pruritus. Each of the patients was asked to verbally assess the intensity of pruritus over the past week on a scale of 0 to 10. It was explained in each case that “0” means complete absence of itching and “10” means the most intense itching the patient can imagine.

In Part II, archival FFPE biopsy specimens from 14 individuals with histologically confirmed CLP was used for immunohistochemical assessment. FFPE biopsy specimens of healthy skin in non-sun-exposed areas from 11 gender- and age-matched healthy subjects undergoing plastic surgery served as control samples.

The research was in accordance with the Declaration of Helsinki and was approved by the local Institutional Review Board. Each subject signed the informed consent form before entering the study.

### 2.2. Serum Level of IL-17

In Part I, approximately 9 ml of venous blood was collected from each study participant (52 patients with CLP and 27 healthy volunteers). The serum obtained after centrifugation was stored at -70°C until all samples were collected. Serum concentration of IL-17 was determined by enzyme-linked immunosorbent assay (ELISA) using the Quantikine® ready ELISA kit (R&D Systems®, USA) Human IL-17. The concentration of IL-17 in serum samples was quantified by determining its optical density using an EPOCH microplate spectrophotometer (BioTEK® Instruments, Inc., USA). The measurement was carried out at a wavelength of 450 nm and a reference wavelength of 540 nm. The value of IL-17 concentration for individual samples was determined on the basis of a standard linear logarithmic curve, automatically created using Gen5® software.

### 2.3. Tissue Expression of IL-17

The immunohistochemical assay was performed using mouse anti-human IL-17 polyclonal antibodies (dilution 0.25 *μ*g; catalogue number PA1-84183; Invitrogen, USA). All determinations were made in accordance with the manufacturer's protocol, with positive and negative control stainings. Visualization was carried out using the Dako RDS AP kit (Dako Real Detection System Alkaine Phosphotase Real Rabbit/Mouse, catalogue number K5005). The preparations were evaluated in a Zeiss Axio Imager A2 light microscope using a video track and Zeiss AxioVision software. The expression was considered to be positive when cell cytoplasmic or membrane immunoreactivity was observed. Positively stained cells were counted in 10 fields of view for each preparation at 200x magnification, and the mean value for each preparation was calculated.

### 2.4. Statistical Analysis

The obtained results were subjected to appropriate statistical analysis. The Shapiro-Wilk test was used to determine the normality of variable distribution. The differences between the groups were determined using Student's *t*-test (for continuous variables showing normal distribution) or the Mann-Whitney *U* test (for continuous variables not showing normal distribution). The chi-square test (with the Yates correction test, where appropriate) and the Fisher exact test were used to compare categorical variables. The relationships between quantitative variables were tested using Spearman's correlation test. Two-sided *p* values were calculated. *p* value < 0.05 was considered statistically significant. All statistical calculations were made using Statistica® software, version 12.0 (Statsoft Poland).

## 3. Results

### 3.1. Characteristics of Patients with CLP

Detailed characteristics of patients with CLP, with regard to gender, are presented in Table 2. Of 52 patients, 37 individuals had isolated papular LP, 2 had isolated hypertrophic LP, 10 had coexisting papular and hypertrophic variants, 1 had coexisting papular and bullous LP, 1 had papular and linear LP, and 1 had papular and pigmented variant of CLP. Oral mucous membranes were the most frequent coexisting location of LP; 28 (53.85%) patients presented with reticular variant of OLP. Genital mucous membranes were involved in 4 (7.69%) patients. Fourteen (26.92%) patients had concomitant nail involvement and 2 (3.85%) patients presented with lichen planopilaris.

Men (*n* = 22) with CLP statistically significantly more often smoked cigarettes compared to women (*n* = 30) with CLP (*p* = 0.03). In addition, oral mucosal involvement was significantly more frequent in men than in women with CLP (*p* = 0.0088). No significant differences were found in terms of age (*p* = 0.24), BMI (*p* = 0.4), age at onset of CLP (*p* = 0.22), duration of the disease (*p* = 0.86), duration of current exacerbation (*p* = 0.91), prevalence of pruritus (*p* = 0.66), severity of pruritus according to the NRS scale (*p* = 0.44), and body surface area involved with LP (*p* = 0.83).

### 3.2. Serum Concentration of IL-17

**Figure 1:**
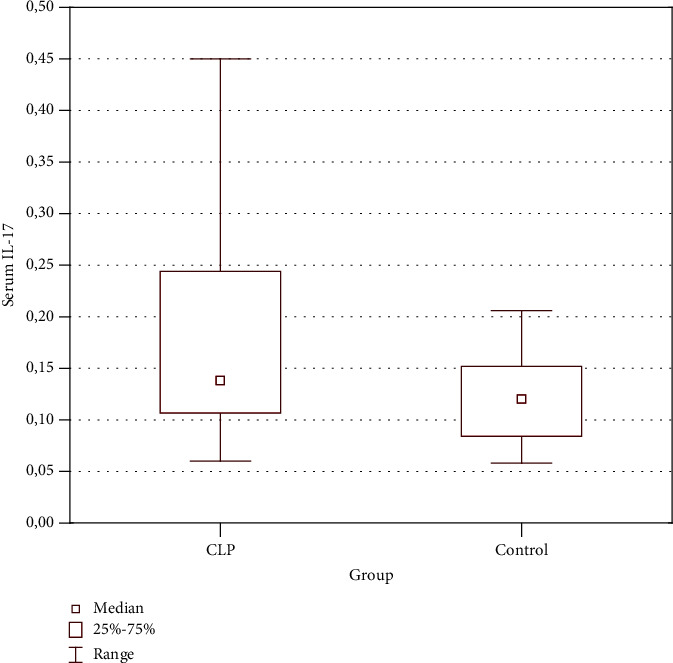
Increased serum level of IL-17 in patients with CLP compared to controls (*p* = 0.025).

The evaluation was performed in 52 patients with CLP and 27 age- and sex-matched healthy blood donors. The serum level of IL-17 was significantly elevated in patients with CLP, compared with healthy volunteers (0.218 ± 0.221 ng/ml versus 0.126 ± 0.058 ng/ml; *p* = 0.025) (Figure 1). The potential associations between serum level of IL-17 and individual clinical parameters were analyzed. There were no differences between serum concentration of IL-17 in men versus women (0.17 ± 0.108 ng/ml and 0.253 ± 0.273 ng/ml, respectively; *p* = 0.34) and smokers versus nonsmokers (0.224 ± 0.194 ng/ml and 0.212 ± 0.194 ng/ml, respectively; *p* = 0.93). Level of IL-17 was also independent of the age of patients (*p* = 0.77; *r* = 0.04), BMI (*p* = 0.26; *r* = 0.16), age at the time of onset of CLP (*p* = 0.84; *r* = 0.03), duration of the disease (*p* = 0.78; *r* = −0.04), duration of the current exacerbation (*p* = 0.49; *r* = −0.1), and the area of the skin affected by the lesions (*p* = 0.66; *r* = 0.06). The presence of pruritus was independent of the serum level of IL-17 (0.207 ± 0.215 ng/ml in patients complaining of itching versus 0.285 ± 0.265 ng/ml in patients negating the occurrence of pruritus; *p* = 0.46). There was also no association between the severity of pruritus according to the NRS scale and the serum level of IL-17 (*p* = 0, 66; *r* = −0.06). Patients with concomitant mucosal and cutaneous involvement had comparable serum concentration of IL-17 to patients with isolated CLP (0.214 ± 0.172 ng/ml and 0.223 ± 0.271 ng/ml, respectively; *p* = 0.46). Similarly, no statistically significant differences were found between patients with coexisting nail involvement and patients without nail lesions (0.156 ± 0.135 ng/ml and 0.241 ± 0.243 ng/ml, respectively; *p* = 0.1).

### 3.3. IL-17 Immunoexpression in CLP Lesions and Controls

**Figure 2:**
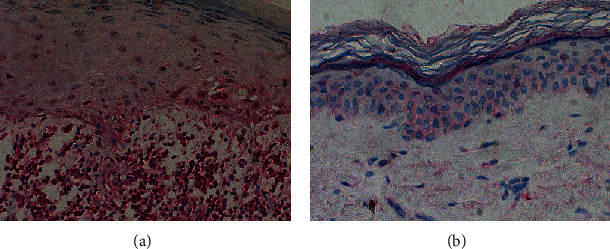
Representative immunohistochemical stainings of IL-17 expression: (a) high number of cells showing positive staining in CLP lesions and (b) single cells showing positive staining in a healthy skin. Magnification, ×200.

Representative expression of IL-17 in specimens of CLP and normal controls is presented in Figure 2. The quantitative evaluation of IL-17 immunoexpression in CLP lesions and controls is shown in Table 3.

**Figure 3:**
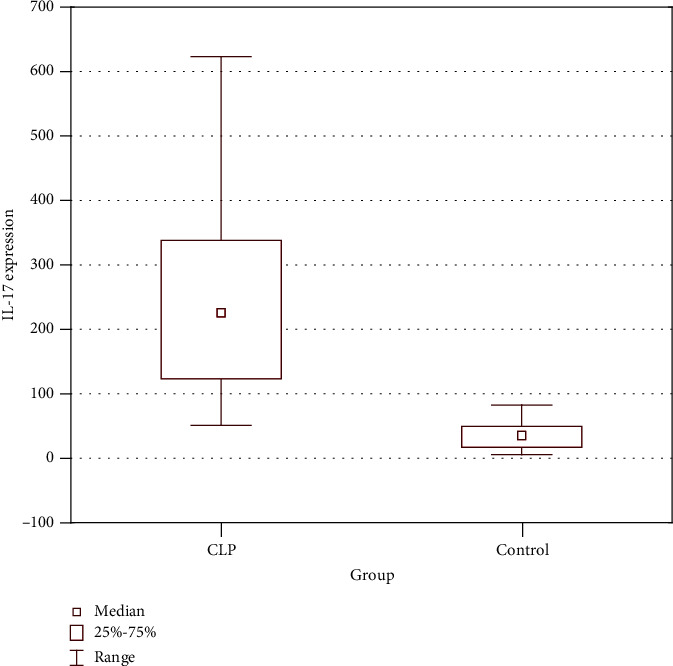
Increased number of cells expressing IL-17 in CLP lesions compared to a healthy skin (*p* < 0.001).

Expression of IL-17 in tissue samples was presented as the mean number of positively stained cells in the field of view ± SD. The average number of cells showing expression of IL-17 in a normal skin was relatively low. In CLP lesions, the average number of cells expressing IL-17 was 247.11 ± 158.05 and in a healthy non-sun-exposed skin—36.57 ± 24.9. The number of cells showing IL-17 expression was significantly higher in CLP lesions compared to healthy controls (*p* < 0.001) (Figure 3).

## 4. Discussion

The potential role of IL-17 in CLP was initially investigated by Shaker and Hassan [22], who measured the serum level of IL-17 in 30 patients with CLP and compared it to 20 healthy controls. To the best of our knowledge, this is the only study in the English language literature so far that focused on the serum concentration of IL-17 in patients with cutaneous variant of LP. The authors found significantly higher level of IL-17 in patients with CLP when compared to healthy volunteers. The patients included in the study by Shaker and Hassan were younger (42.7 ± 15.4 years) than the present study population, had similar disease duration (3.3 ± 5.4 years), but lower percentage of the affected skin surface (9.6 ± 8.0%). Shaker and Hassan did not find statistically significant differences in the serum level of IL-17 between males and females and did not observe significant correlation between serum IL-17 level and patient age, disease duration, or extent of skin involvement. Unfortunately, the authors did not specify the percentage of patients with concomitant oral LP involvement.

In our study, we found significantly higher levels of IL-17 in patients with CLP compared with healthy controls. Similar to the findings from the aforementioned research, serum IL-17 concentrations were independent from patient age, gender, disease duration, and extent of skin involvement. In our study, we devoted more attention to evaluation of the presence and severity of pruritus. Itchiness turned out to be a significant complaint, reported by 86.54% of patients. Nevertheless, IL-17 does not appear to be a mediator of pruritus in CLP, as its level did not show any relationship with the presence or severity of itching. We also evaluated the presence of concomitant LP involvement in other locations. In our study, 26.92% of patients had LP limited to the skin and 53.85% of patients had coexisting OLP. Nevertheless, we did not find any statistically significant relationship between the serum level of IL-17 and the presence of mucosal or nail involvement with LP.

Moreover, we found high expression of IL-17 in CLP lesions. Still, it remains unclear which immune cells are the main source of IL-17 in CLP. IL-17 is referred to as the signature cytokine of Th17 cells. Interestingly, neutrophils and mast cells are considered to be a relevant source of IL-17 in psoriasis [4]. Recently, there is a growing interest in IL-17-producing CD8(+) T cells, so-called Tc17 cells, which are supposed to play a key role in autoimmune conditions. CD8(+) T cells are potential candidates to be the main source of IL-17 in OLP, where they constitute the majority of the inflammatory infiltrate in the lesional mucous membrane [3]. Solimani et al. [25] performed multiplex immunohistochemistry analysis in a lesional skin of CLP and identified CD4(+) and CD8(+) cells showing positive expression of IL-17, which points that both Th17 and Tc17 cells may be implicated in the pathogenesis of CLP.

As LP often poses a therapeutic challenge due to the refractory course, there is a need for novel treatment strategies that would give hope to patients suffering from the most recalcitrant and severely itchy variants of the disease. Monoclonal antibodies blocking the IL-17 pathway (secukinumab, ixekizumab, and brodalumab) are currently successfully used in the treatment of psoriasis and psoriatic arthritis. Recently, Solimani et al. [25] reported a case series of five patients with severe OLP, two of whom had coexisting cutaneous involvement, successfully treated with either secukinumab (anti-IL-17), ustekinumab (ant-IL-12/23), or guselkumab (anti-IL-23). This report points that the closely related IL-23 and IL-17 pathways may be implicated in the development of OLP. As the two patients with concomitant cutaneous variant of LP, treated with secukinumab, achieved significant improvement of skin condition in the form of rapid shift from erythematous papules and plaques to postinflammatory hyperpigmentation, it can be assumed that IL-17 may represent a major therapeutic target in CLP.

Our findings suggest that IL-17 may be a critical mediator in the pathogenesis of CLP. Further studies are needed to evaluate the exact role and origin of IL-17 in CLP and the potential role of monoclonal antibodies blocking the IL-17 pathway in the treatment of persistent variants of the disease.

## Figures and Tables

**Table 1:** Demographic characteristics of the study group.

Characteristics	Patients with CLP (*n* = 52)	Control group (*n* = 27)	*p* value
Age in years (mean ± SD)	51.6 ± 15.55	45.59 ± 10.15	*p* = 0.07
Gender (*n* (%))			
Males	22 (42.3)	15 (55.56)	*p* = 0.26
Females	30 (57.7)	12 (44.44)	

**Table 2:** Clinical characteristics of patients with CLP, with regard to gender.

Clinical characteristics	Patients with CLP (*n* = 52)	Men with CLP (*n* = 22)	Women with CLP (*n* = 30)	*p* value
Age in years (mean ± SD)	51.6 ± 15.55	48.59 ± 16.98	53.8 ± 14.29	0.24
BMI in kg/m^2^ (mean ± SD)	26.92 ± 5.15	26.22 ± 3.91	27.44 ± 5.92	0.4
Cigarette smokers (*n* (%))	24 (46.15)	14 (63.64)	10 (33.33)	*0.03*
Age at CLP onset in years (mean ± SD)	48.5 ± 15.74	45.38 ± 17.58	50.79 ± 14.11	0.22
Disease duration in years (mean ± SD)	3.15 ± 6.89	3.36 ± 7.9	3.0 ± 6.22	0.86
Duration of current exacerbation in months (mean ± SD)	8.29 ± 16.33	8.62 ± 22.92	8.06 ± 9.85	0.91
Presence of pruritus (*n* (%))	45 (86.54)	18 (81.81)	27 (90)	0.66
Intensity of pruritus (NRS) (mean ± SD)	5.25 ± 3.03	4.68 ± 3.24	5.53 ± 2.9	0.44
Body surface area (%) (mean ± SD)	13.23 ± 15.35	12.67 ± 14.86	13.63 ± 15.93	0.83

Other locations (*n* (%))				
Oral mucous membranes	28 (53.85)	17 (77.27)	11 (36.67)	*0.0088*
Genital mucous membranes	4 (7.69)	1 (4.55)	3 (10)	0.84
Nails	14 (26.92)	6 (27.27)	8 (26.67)	0.96
Scalp	2 (3.85)	0 (0)	2 (6.67)	0.61

Number of locations (*n* (%))				
Single	14 (26.92)	2 (9.09)	12 (40)	
Two	30 (57.69)	17 (77.27)	13 (43.33)	
Three	7 (13.46)	3 (13.64)	4 (13.33)	
Four	1 (1.92)	0 (0)	1 (3.33)	

Clinical subtype of CLP (*n* (%))				
Papular	50 (96.15)	20 (90.9)	30 (100)	0.34
Hypertrophic	12 (23.08)	8 (36.36)	4 (13.33)	0.11
Bullous	1 (1.92)	0 (0)	1 (3.33)	0.86
Linear	1 (1.92)	1 (4.55)	0 (0)	0.88
Pigmented	1 (1.92)	0 (0)	1 (3.33)	0.86

Serum level of IL-17 (ng/ml) (mean ± SD)	0.218 ± 0.221	0.17 ± 0.108	0.253 ± 0.273	0.34

**Table 3:** Quantitative comparison of the immunoexpression of IL-17 in tissue specimens of CLP lesions and healthy controls (values expressed as the number of positively stained cells).

	CLP lesions (*n* = 14)	Control (normal skin) (*n* = 11)	*p* value
IL-17 expression			
Mean	247.11	36.57	*<0.001*
SD	158.05	24.9	
Range	51.2–623	5.7–82.6	

## Data Availability

The data used to support the findings in the presented study are available from corresponding author upon request.
